# Microsatellite markers for identification and parentage analysis in the European wild boar (*Sus scrofa*)

**DOI:** 10.1186/1756-0500-5-479

**Published:** 2012-09-03

**Authors:** Vânia Costa, Javier Pérez-González, Pedro Santos, Pedro Fernández-Llario, Juan Carranza, Attila Zsolnai, István Anton, József Buzgó, Gyula Varga, Nuno Monteiro, Albano Beja-Pereira

**Affiliations:** 1 Centro de Investigação em Biodiversidade e Recursos Genéticos da Universidade do Porto (CIBIO-UP), Rua Padre Armando Quintas 7, 4485-661, Vairão, Portugal; 2 Biology and Ethology Unit, University of Extremadura, 10071, Cáceres, Spain; 3 Instituto de Ciências Agrárias e Ambientais Mediterrânicas (ICAAM), Universidade de Évora, Herdade da Mitra, 7002-554, Évora, Portugal; 4 Research Institute for Animal Breeding and Nutrition, H-2053, Herceghalom, Hungary; 5 University of Kaposvár, H-7400, Kaposvár, Hungary; 6 SEFAG Forest Management and Wood Industry Share Company, Kaposvár, Hungary; 7 Department of Animal & Plant Sciences, University of Sheffield, S10 2TN, Sheffield, UK; 8 Ungulate Research Unit, CRCP, University of Córdoba, 14071, Córdoba, Spain; 9 CEBIMED, Faculty of Health Sciences, University Fernando Pessoa, R. Carlos da Maia, 296, 4200-150, Porto, Portugal

**Keywords:** Sus scrofa, Parentage assignment, Individual identification, Microsatellite markers, Wild boar

## Abstract

**Background:**

The wild boar (*Sus scrofa*) is among the most widespread mammal species throughout the old world. Presently, studies concerning microsatellites in domestic pigs and wild boars have been carried out in order to investigate domestication, social behavior and general diversity patterns among either populations or breeds. The purpose of the current study is to develop a robust set of microsatellites markers for parentage analyses and individual identification.

**Findings:**

A set of 14 previously reported microsatellites markers have been optimized and tested in three populations from Hungary, Portugal and Spain, in a total of 167 samples. The results indicate high probabilities of exclusion (0.99999), low probability of identity (2.0E^-13^ – 2.5E^-9^) and a parentage assignment of 100%.

**Conclusions:**

Our results demonstrate that this set of markers is a useful and efficient tool for the individual identification and parentage assignment in wild boars.

## Background

The wild boar, *Sus scrofa*, is currently one of the most widespread wild mammal species, inhabiting an extensive range of environments [1]. Its domestic form, the pig, is of economic importance, and the present day varieties are the result of multiple domestication events that occurred in different regions [2]. Many studies have employed a wide array of genetic markers aimed at inferring domestication, migration processes, and examining patterns of genetic diversification for both domestic and wild forms. Nevertheless, the peculiar reproductive behaviour and mating system of the wild boar have been poorly characterized [3]. While some authors state that litters are sired by a single father [4], others suggest the existence of multiple paternity [3,5], thus pointing to either a polygynous (one male mating with several females) or polygynandrous mating system (both males and females mate with distinct individuals).

During the last decades, advances in genome sequencing and mapping studies have reported thousands of polymorphic neutral markers, such as microsatellites, which have proven to be powerful tools for parental and kinship analysis. In this study, we have chosen to use microsatellite loci given their higher variability (e.g., on average expected heterozygosity (He) is higher than 0.6) and consequently increased power for parentage assignment when compared to the same number of bi-allelic markers such as SNPs [6,7]. Thus, we constructed a panel of polymorphic microsatellite based on two main criteria: 1) suitability to be combined and amplified in-group (plexes) and 2) an average He value higher than 0.6. We believe that the development and validation of such a panel of markers will provide a valuable tool to assess parental and kinship relationships, thus allowing considerable improvement for studies of mating behavior of both domestic and wild pigs.

## Findings

**Figure 1:**
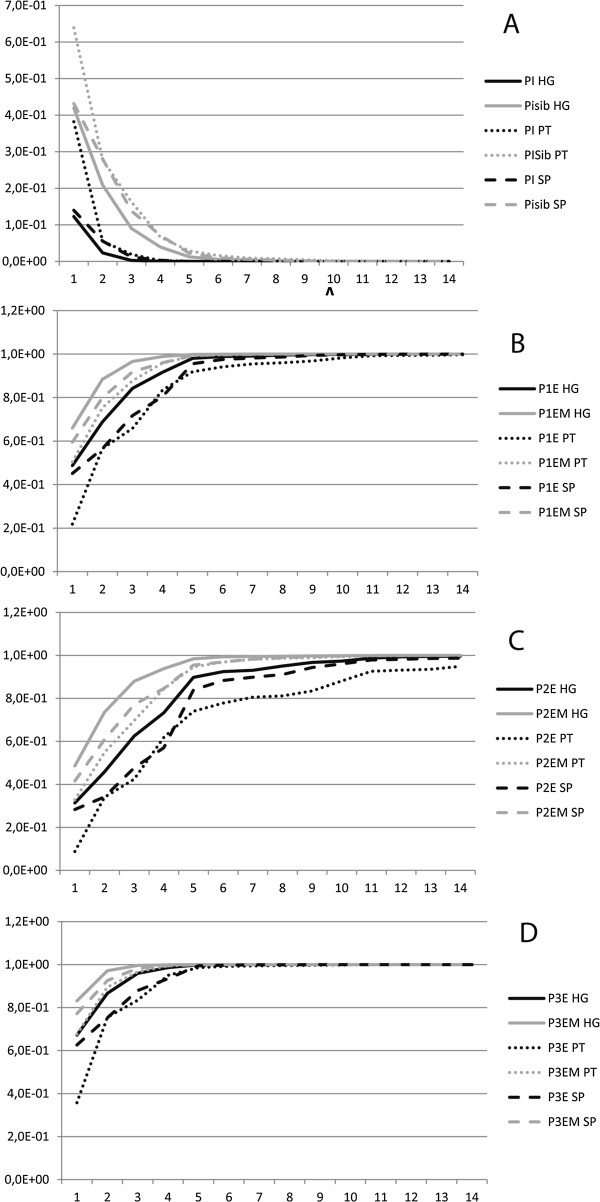
Graphical representation of the identity and exclusion probabilities for the combination of selected markers: (A) probability of identity (PI) and probability of identity when related individuals are included in the sample (PIsibs); (B) probability of exclusion when both parents are unknown (P1X) and its maximization (P1XM); (C) probability of exclusion when one of the parents is known (P2X) and its maximization (P2XM); and (D) probability of exclusion for two putative parents (P3X) and its maximization (P3EM); Population codes: HG – Hungary, PT – Portugal, SP - Spain.

Our results confirm the effectiveness of the microsatellite panel for establishment of parentage in wild boars. The probability of identity (PI, the probability of two independent samples having the same identical genotype), using all 14 microsatellites, results in values as low as 2.0E^-13^, 2.5E^-9^ and 1.2E^-11^ for the Hungarian, Portuguese and Spanish populations, respectively. Although the values obtained using all the 14 loci provide low values, similar PI values are obtained by combining only five loci, in the case of the Hungarian and Spanish populations, and six loci in the case of the Portuguese populations (Figure 1). The probability of identity when related individuals are included on the samples (PISibs) is also low, attaining the minimum probabilities for the 14 loci combination of 1.0E^-5^, 1.6E^-4^ and 3.1E^-5^ for Hungary, Portugal and Spain, respectively. The probability of exclusion when both parents are unknown (P1X), when one of the parents is known (P2X) and when the parents are putative (P3X), the maximum probability was different for each of the three populations (Figure 1).

Loci informativeness, heterozygosity levels and exclusion probability values are depicted in Table 1. The probability of finding null alleles is generally negligible, with the exception of three loci (Sw24, S0101, Sw857) in the Hungarian population and one locus on the Spanish population (S0226). Our analyses suggest that in both Hungarian and Spanish populations no microsatellite had significant deviations from Hardy-Weinberg proportions, following Bonferroni correction. In the Portuguese population, however, we found deviations on loci SW857 and SW72 (p < 0.003). Also, after Bonferroni corrections, only four out of 91 combinations exhibited significant deviations from a random association between alleles at different loci.

**Table 1:** Summary statistics results for the three populations (HG – Hungary, PT – Portugal, SP – Spain) using the 14 microsatellite loci: number of individuals analyzed (N), number of alleles of each locus (k), observed heterozygosity (Hobs), expected heterozygosity (HExp), average non-exclusion probability for the first parent (NE-1P), average non-exclusion probability for the second parent (NE-2P), average non-exclusion probability for a candidate parent pair (NE-PP), average non-exclusion probability for identity of two unrelated individuals (NE-I), average non-exclusion probability for identity of two siblings (NE-SI), and estimated null allele frequency (F(null))

		**Sw24**	**S0155**	**Sw936**	**Sw2410**	**S005**	**Sw632**	**Sw857**	**S0226**	**Sw72**	**Sw240**	**S0068**	**S0101**	**Sw122**	**Sw2008**
**N**	**HG**	49	47	49	49	46	49	49	47	47	48	46	48	43	47
	**PT**	72	72	72	72	69	71	71	70	72	72	66	72	72	72
	**SP**	46	46	46	46	43	45	46	46	46	46	43	46	46	46
**k**	**HG**	6	6	7	6	14	7	3	4	5	4	8	8	6	3
	**PT**	4	4	4	6	10	5	5	3	3	5	9	5	5	3
	**SP**	5	4	5	4	12	4	5	4	6	5	9	6	4	4
**Hobs**	**HG**	0.9	0.5	0.6	0.65	0.85	0.75	0.5	0.75	0.833	0.65	0.895	1	0.733	0.444
	**PT**	0.415	0.61	0.537	0.659	0.718	0.5	0.575	0.268	0.415	0.732	0.861	0.39	0.317	0.585
	**SP**	0.773	0.364	0.636	0.636	0.909	0.762	0.545	0.409	0.773	0.773	0.7	0.591	0.545	0.636
**Hexp**	**HG**	0.741	0.615	0.715	0.713	0.901	0.679	0.396	0.737	0.754	0.596	0.868	0.817	0.791	0.452
	**PT**	0.418	0.71	0.511	0.749	0.706	0.536	0.501	0.259	0.494	0.712	0.776	0.369	0.326	0.662
	**SP**	0.723	0.411	0.634	0.606	0.906	0.721	0.508	0.504	0.784	0.722	0.827	0.537	0.624	0.532
**NE-1P**	**HG**	0.686	0.789	0.693	0.712	0.385	0.736	0.925	0.71	0.67	0.817	0.475	0.568	0.631	0.903
	**PT**	0.912	0.723	0.872	0.663	0.681	0.853	0.876	0.967	0.881	0.718	0.623	0.932	0.945	0.786
	**SP**	0.717	0.918	0.796	0.82	0.375	0.727	0.868	0.877	0.632	0.704	0.549	0.848	0.809	0.857
**NE-2P**	**HG**	0.513	0.608	0.506	0.533	0.237	0.556	0.826	0.54	0.49	0.652	0.308	0.391	0.453	0.805
	**PT**	0.781	0.555	0.787	0.486	0.492	0.718	0.777	0.877	0.793	0.55	0.445	0.812	0.818	0.639
	**SP**	0.549	0.793	0.649	0.691	0.23	0.562	0.713	0.772	0.454	0.526	0.373	0.686	0.673	0.701
**NE-PP**	**HG**	0.328	0.408	0.3	0.341	0.083	0.358	0.721	0.369	0.301	0.475	0.137	0.205	0.269	0.696
	**PT**	0.642	0.381	0.677	0.3	0.277	0.566	0.659	0.787	0.684	0.374	0.257	0.686	0.683	0.49
	**SP**	0.374	0.662	0.486	0.543	0.081	0.394	0.545	0.649	0.272	0.337	0.19	0.507	0.521	0.534
**NE-I**	**HG**	0.123	0.19	0.12	0.133	0.025	0.15	0.434	0.133	0.109	0.22	0.042	0.069	0.093	0.389
	**PT**	0.382	0.144	0.35	0.109	0.114	0.284	0.344	0.576	0.36	0.141	0.09	0.437	0.472	0.193
	**SP**	0.14	0.398	0.213	0.248	0.024	0.146	0.289	0.34	0.092	0.128	0.063	0.262	0.231	0.272
**NE-SI**	**HG**	0.419	0.497	0.431	0.436	0.317	0.456	0.665	0.424	0.411	0.514	0.338	0.369	0.391	0.627
	**PT**	0.639	0.435	0.585	0.407	0.43	0.556	0.589	0.766	0.596	0.433	0.39	0.677	0.707	0.472
	**SP**	0.432	0.649	0.493	0.516	0.313	0.434	0.574	0.589	0.39	0.429	0.363	0.553	0.503	0.558
**F(null)**	**HG**	−0.1106	0.03	0.0296	0.0586	0.0341	−0.0317	0.0208	0.0012	−0.0237	−0.0763	−0.0072	−0.0394	0.0259	0.0825
	**PT**	0.0115	0.0105	0.1312	0.0755	−0.0173	0.0174	−0.0431	−0.0293	−0.0235	−0.0014	−0.0468	0.0618	0.0377	0.1476
	**SP**	−0.0673	−0.0396	−0.0143	−0.0969	−0.0029	0.027	−0.0549	−0.0013	0.0021	−0.0128	0.0391	−0.1238	0.0292	−0.0785

Assignment probabilities, using COLONY v.2.0, revealed a maternity probability of 1 for all mother/litter combinations. Nevertheless, none of the males captured within the same area of the pregnant females was found to sire any offspring. Nonetheless, the paternal genotypes were inferred and revealed that ten of the litters had one exclusive father while two litters from Hungary, two from Spain and one in Portugal presented genotypes consistent with the simultaneous occurrence of at least two different fathers (multi-paternity). The calculated full- and half-sibs probabilities were also consistent with these findings.

## Conclusions

Our results support the usefulness of the described set of microsatellites as a valuable tool for parentage analysis in the wild boar, as the individual identification and power of exclusion levels reveal high power and accuracy (Figure 1). Indeed, due to the high information content (Table 1) of some of the microsatellite markers, it is even possible to obtain a high precision in individual identification and parentage assignment with a subset of only 6 markers.

Although many microsatellites have been described both for domestic pigs and wild boars, some of them are not suitable for assignments since 1) they are not sufficiently informative, thus requiring a higher number of markers to satisfactory results, or 2) present technical constrains, such as difficulty on amplification, scoring or poor performance in a multiplex setting. The microsatellite loci here reported were especially selected to overcome those technical limitations, providing diversity levels which are comparable to the molecular tools reported in previous works concerning parentage analysis [5,8].

The selected microsatellites present high levels of informativeness, and null allele frequencies were found to be above 10%, even if only in a restricted number of loci, in Hungary and Spain (Table 1). Nevertheless, it should be taken into account that the estimation of null alleles is highly influenced by the sampling of substructured populations. Indeed, previous studies also detected similar deviations in other wild boar populations [4,9], although in our case these deviations were only found in the Portuguese population. Since our data quality control rules out the possibility of genotyping errors as the possible source of these deviations, the most probable cause for the unbalance on the Portuguese wild boar population may be related to recent demographic fluctuation that certainly may have left a strong mark on the population genetic structure. Nonetheless, the similarity in the expected heterozygosity values (Table 1) obtained in these loci when compared with those reported for different species [9,10], confirms the reliability of this set of markers and its power of resolution for parentage assignment.

Finally, we believe that the inability to detect any putative father within our samples might result from distinct, non-exclusive, causes ranging from 1) insufficient male sampling, 2) the specificity of the wild boar mating behavior where males do not guard females once copulation ends and 3) the indirect consequences of the hunting process which uses dogs and human noise to direct animals towards a group of hunters, thus unbalancing the opportunity to capture solitary adult males or groups of females [5,11]. Even though the actual father was not retrieved from our own samples, we were nevertheless able to infer the potential fathers’ genotypes using the genotype reconstruction implemented in COLONY v. 2.0 [12,13], an invaluable tool for the determination of the wild boar mating system.

## Methods

A total of 167 tissue samples were collected from wild boars hunted in Portugal (36 males and 5 pregnant females bearing 31 offspring), Spain (17 males and 5 pregnant females bearing 24 offspring) and Hungary (15 males and 5 pregnant females bearing 29 offspring). All samples used in this work came from dead hunted animals. The dead of the animals did not result for this work, but from legal game hunting activities. Samples were collected, at the end of the day, from dead hunted animals after veterinary inspection. The tissue samples were extracted with JETQUICK Tissue DNA Spin Kit (Genomed, GmbH) according to the manufacturer’s protocol. A total of 27 fluorescent-labeled microsatellite markers were initially tested with a small panel of wild boar samples (n = 16), after a thorough selection based on available bibliographic data (ref. [14-19]; Table 2). Some of the analyzed markers were discarded either due to low levels of polymorphism (less than 3 alleles), lack of multiplex assay robustness, insufficient information content and difficulty to amplify or score. Finally, a total of 14 loci were chosen and optimized in two multiplex panels each containing 7 microsatellites (Table 2). PCR amplification were carried in two independent reactions with the same procedure – a total volume of 10 μl containing 10 ng of genomic DNA, 10 mM of primer mix (Table 2), Qiagen Multiplex PCR Master Mix (QIAGEN GmbH, Hilden) and water. The reaction conditions were as follow: (1) an initial denaturation at 95°C for 15 minutes; 2) 10 cycles of 95°C for 30 seconds, 60-56°C (ΔT −0.5°C) for 90 seconds and 72°C for 45 seconds; (3) 22 cycles of 95°C for 30 seconds, 56°C for 90 seconds and 72°C for 45 seconds; (4) 8 cycles of 95°C for 30 seconds, 53°C for 90 seconds and 72°C for 45 seconds, and (5) a final extension step on 72°C for 30 minutes. The samples were then tested in 2% agarose gel and their concentration normalized. 

**Table 2:** Characterization of the STR primer sequence, fluorescent dye used, size range, chromosome location and reference

	**Locus**	**Primer Sequence (5'-3')**	**Dye**	**Size Range**	**Chr.**	**Reference**
**Plex1**	**Sw24**	F: CTTTGGGTGGAGTGTGTGC	FAM	113-137	17	[14]
		R: ATCCAAATGCTGCAAGCG				
	**S0155**	F: TGTTCTCTGTTTCTCCTCTGTTTG	NED	167-183	1	[15]
		R: AAAGTGGAAAGAGTCAATGGCTAT				
	**Sw936**	F: TCTGGAGCTCGCATAAGTGCC	PET	115-133	15	[14]
		R: GTGCAAGTACACATGCAGGG				
	**Sw2410**	F: ATTTGCCCCCAAGGTATTTC	VIC	122-140	A	[16]
		R: CAGGGTGTGGAGGGTAGAAG				
	**S0005**	F: TCCTTCCCTCCTGGTAACTA	NED	223-275	5	[16]
		R: GCACTTCCTGATTCTGGGTA				
	**Sw632**	F: TGGGTTGAAAGATTTCCCAA	VIC	177-197	7	[14]
		R: GGAGTCAGTACTTTGGCTTGA				
	**Sw857**	F: TGAGAGGTCAGTTACAGAAGACC	PET	168-178	14	[14]
		R: GATCCTCCTCCAAATCCCAT				
**Plex2**	**S0226**	F: GCACTTTTAACTTTCATGATACTCC	PET	202-212	2	[17]
		R: GGTTAAACTTTTNCCCCAATACA				
	**Sw72**	F: ATCAGAACAGTGCGCCGT	VIC	119-133	3	[14]
		R: TTTGAAAATGGGGTGTTTCC				
	**Sw240**	F: AGAAATTAGTGCCTCAAATTGG	FAM	112-130	2	[14]
		R: AAACCATTAAGTCCCTAGCAAA				
	**S0068**	F: AGTGGTCTCTCTCCCTCTTGCT	VIC	246-280	13	[18]
		R: CCTTCAACCTTTGAGCAAGAAC				
	**S0101**	F: GAATGCAAAGAGTTCAGTGTAGG	NED	216-238	7	[19]
		R: GTCTCCCTCACACTTACCGCAG				
	**Sw122**	F: CAAAAAAGGCAAAAGATTGACA	PET	127-143	6	[14]
		R: TTGTCTTTTTATTTTGCTTTTGG				
	**Sw2008**	F: CAGGCCAGAGTAGCGTGC	NED	116-122	11	[16]
		R: CAGTCCTCCCAAAAATAACATG				

The multiplex products were added to a mixture of Hi-Di™ formamide and size standard (Gene Scan™ 500 LIZ size standard) and run in a 3130 XL Genetic Analyzer (Life Technologies) sequencer. GENE MAPPER v4.0 (Applied Biosystems, USA) software was used to analyze the resulting electropherograms in order to identify the obtained alleles. Data quality assessments were scattered along the process either in the form of negative controls (e.g., to exclude contamination problems), or in a final step consisting of a re-amplification and genotyping of randomly chosen samples (10%) to insure a perfect match in the obtained results [20].

All the data analyses were performed independently for each population and with the exception of maternity and paternity assignment and null alleles, were accomplished using only the adult individuals. The software GENALEX v. 6.41 [21] was used to calculate the deviations from Hardy-Weinberg Equilibrium proportions (HWE), the probability of identity and the power of exclusion for the loci combinations when both parents are known, when one of the parents is known and for two putative parents. Cervus v. 3.0 [22] software was used to analyze the number of alleles, observed and expected heterozygosity, combined non-exclusion probabilities (for the first parent and second parent, parent pair, identity and siblings identity) and the estimated null allele frequency (including mothers and offspring). Genepop v.1.2 [23] software was utilized to calculate the gametic disequilibrium (1000 dememorization steps, 10 batches, 1000 interactions per batch) between alleles of different loci and for each population individually. A maximum-likelihood method implemented in COLONY v. 2.0 [12,13] was then used to calculate the assignment probabilities in parentage and sib-ship analyses.

## Competing interests

The authors have no competing interests to declare.

## Authors’ contributions

VC carried out the microsatellite genotyping and drafted the manuscript. JP-G performed part of the statistical tests and sampling. PS, PF-L, JC, JB, GV, AI, AZ, organized the sampling campaigns. AB-P, PS, and NM participated in the design of the study and performed the statistical analysis. AB-P conceived of the study, and participated in its design and coordination and helped to draft the manuscript. All authors read and approved the final manuscript.
